# A distinct variant of papillary thyroid carcinoma indicating familial adenomatous polyposis (FAP): a case report and brief review

**DOI:** 10.1186/s13104-015-1736-1

**Published:** 2015-12-17

**Authors:** Nishantha Liyanapathirana, Sanjeewa Anuruddha Seneviratne, Dharmabandhu Nandadeva Samarasekera

**Affiliations:** University Surgical Unit, National Hospital of Sri Lanka, Colombo, 10 Sri Lanka

**Keywords:** Familial adenomatous polyposis, Papillary thyroid carcinoma, Cribriform morular variant

## Abstract

**Background:**

Familial adenomatous polyposis (FAP) is an autosomal dominantly inherited intestinal polyposis syndrome with an incidence of about 1/8300 births and accounts for about 1 % of all colorectal cancers. It has a spectrum of extra-intestinal manifestations including thyroid carcinoma which occur in 1–2 % of affected. The cribriform morular variant (CMV) is a rare but distinct histological subtype of papillary thyroid carcinoma (PTC) associated with FAP. Most of the reported cases describe the above entity in the background of well-established FAP. We report a case where both entities presenting simultaneously in a previously undiagnosed patient with FAP without a family history of polyposis.

**Case presentation:**

A 24 year old Asian female presented to the surgical clinic with a goitre of eight months duration and recent onset of altered bowel habits with features of anaemia. She was previously healthy and there was no family history of adenomatous polyposis, colorectal carcinoma or thyroid neoplasms. Colonoscopy revealed large bowel polyposis and fine needle aspiration of thyroid revealed a smear suspicious for malignancy. She underwent total thyroidectomy which revealed CMV PTC. Histology was characterized by a prominent cribriform pattern of growth with interspersed cell clusters arranged as morules along with papillary structures which are the key features of this subtype.

**Conclusion:**

Diagnosis of CMV warrants ruling out of underlying FAP, irrespective of family history or gastrointestinal symptoms.

## Background

Familial adenomatous polyposis (FAP) is an inherited autosomal dominant syndrome which is characterized by innumerable colorectal polyps that have an intrinsic tendency to progress to adenocarcinoma. Extra intestinal manifestations including papillary thyroid carcinoma (PTC) are well described in FAP. The cribriform morular variant (CMV), a distinct and a rare sub type of PTC associated with FAP, has been reported in the literature previously, but in limited numbers. However most of the reported cases describe the above entity in the background of well-established FAP. We report a case where both entities presenting simultaneously in a previously undiagnosed patient with FAP without a family history of polyposis.

## Case report

A 24 year old Asian female presented to the surgical clinic with a goitre of eight months duration and recent onset of altered bowel habits (i.e., increased stool frequency) with features of anaemia. She was otherwise well and there was no family history of adenomatous polyposis, colorectal carcinoma or thyroid neoplasms. Physical examination revealed a multi nodular goitre. Ultrasonically both lobes of the thyroid gland were enlarged with multiple hyper-echoic nodules with central irregularity denoting necrosis. Fine needle aspiration cytology was compatible with a smear suspicious of papillary thyroid malignancy. The patient was biochemically euthyroid and antithyroid antibody status was negative.

Colonoscopy revealed multiple polyps (more than 100) of varying sizes from the caecum extending to the rectum. Histology showed adenomatous polyps with low grade dysplasia rendering the diagnosis of FAP.

**Fig. 1:**
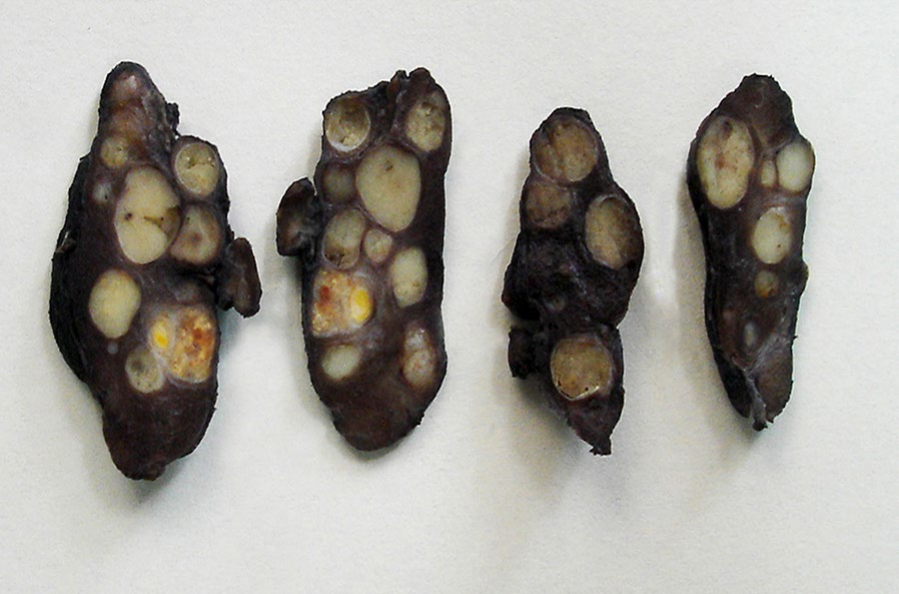
Macroscopic appearance of the tumour displaying multifocality

She underwent a total thyroidectomy with level VI lymph node dissection and the recovery was uneventful. Macroscopy revealed multiple circumscribed whitish tumours of varying sizes in both thyroid lobes (Fig. 1). There were 14 separate tumours in total, the largest measuring 18 × 17 × 14 mm in size. Some of the lesions contained areas of haemorrhages and cystic changes.

**Fig. 2:**
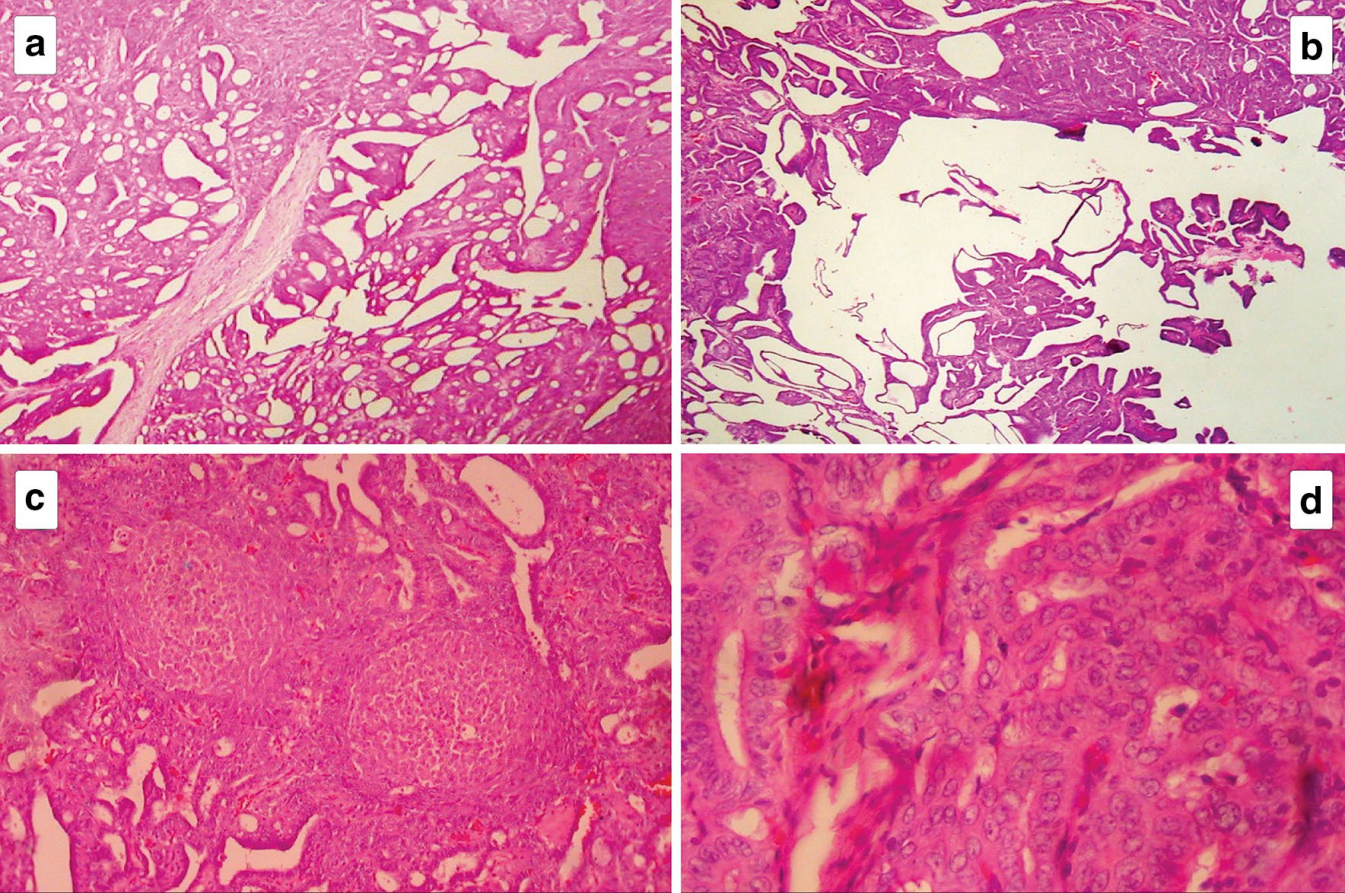
Microscopic appearance of Cribriform morular variant of papillary thyroid carcinoma, showing all under mentioned features in the same tumour. **a** Cribriform growth pattern. **b** Papillary growth pattern. **c** Characteristic whorls of cell nests forming morules. **d** Cytological details—rounded cells with clear nuclei

Microscopically, all the tumours showed predominant features of encapsulated PTC. However, unlike in the usual PTC, a variety of architectural growth patterns including cribriform, solid and trabecular with morule formation were noted along with the papillary structures (Fig. 2). Constituent cells were cuboidal to columnar with amphophilic cytoplasm. Spindle shaped cells were noted in morular structures. Capsular invasion was seen in one tumour but there was no evidence of vascular invasion. The lymph nodes were free of metastases.

The patient was started on high dose thyroxine replacement and referred for radio iodine treatment. Disease involvement of the small bowel was excluded with capsule endoscopy and restorative proctocolectomy is being planned for the colonic polyposis. Colonoscopy screening of her two brothers and parents was found to be negative for FAP.

## Discussion and conclusion

FAP is an autosomal dominantly inherited intestinal polyposis syndrome, which occurs due to a germ line mutation of the adenomatous polyposis coli (APC) gene located on chromosome 5q21. It is characterized by the development of multiple adenomatous polyps throughout the colon which invariably leads to colorectal carcinoma. Although FAP is inherited in an autosomal dominant mode, it is also reported to occur in 25–30 % as “De Novo” [1]. Further it exhibits variable penetrance with marked inter and intra familial variations. Extra intestinal manifestations in FAP includes malignancies such as thyroid and pancreatic cancer, hepatoblastomas, central nervous system tumours (especially medulloblastomas) and various benign tumours such as adrenal adenomas, osteomas, desmoid tumours and dental abnormalities. The relationship between FAP and thyroid carcinoma was first suggested in 1968 [2]. The CMV of PTC was first recognized by Harach et al. [3] as a peculiar form of thyroid carcinoma developing in patients with FAP, and the term CMV was proposed by Cameselle-Teijeiro and Chan in 1999 [6]. It is characterized by female predominance (female/male ratio—17:1) and the mean age of presentation is 30 years. In over 90 % of patients with FAP and PTC, histologic features are compatible with CMV and most exhibit multifocal development [4, 5]. There are reported cases of sporadic CMV of PTC in the absence of FAP, which are often solitary [6, 7].

The CMV has a very unusual histology, characterized by a prominent cribriform pattern of growth with interspersed cell clusters arranged in squamoid islands (morules) [6]. The cribriform areas are composed of anastomosing bars and arches of cells without intervening stroma with the follicular spaces devoid of colloid (Fig. 2a). They are typically formed by cuboidal or tall cells [8].

Morules consists of solid areas where whorls of cell clusters are arranged as nests (Fig. 2c). They are mainly composed of spindle and oval shaped tumour cells. Nuclei of these cells exhibit a characteristic pale staining (“peculiar nuclear clearing”) which differ them from both optically clear nuclei (Orphan Annie appearance) and intranuclear pseudo-inclusions that are more typically seen in PTC [8].

Molecular genetic studies are carried out in suspected FAP using PCR analysis in both the tumour tissue and the patient’s white blood cells. While it detects the presence of APC gene mutation it also confirms whether there is a germline mutation (observed in FAP associated CMV PTC) or a somatic mutation (observed in sporadic cases). However an alternative pathway with aberrant nuclear accumulation of beta-catenin which results from mutation of beta-catenin gene (CTNNB1), substituting the APC mutation has also been observed in some cases of CMV PTC [9]. Although our patient refused to undergo genetic analysis, presence of colonic polyposis and the younger presenting age strongly suggested the possibility of FAP associated CMV of PTC.

Immunohistochemically, the hallmark feature of these tumours which differentiates them from classical PTC is the intra-cytoplasmic and intra-nuclear positivity for beta-catenin. In classical PTC beta-catenin activity is only limited to the cell membranes, which highlights the role of aberrant cellular and nuclear accumulation of beta-catenin resulting from mutant APC or CTNNB1 gene, in triggering this peculiar variant [8].

Distant metastasis is rarely observed in CMV of PTC and the standard treatment consists of total thyroidectomy followed by radioiodine therapy [6, 10]. Good long term prognosis with a five year survival rate of over 90 % and 20 year survival rate of 77 % has been observed in FAP associated CMV PTC [6, 10], which is comparable to classical PTC. This highlights the significance of appreciation of histological features and accurate diagnosis of this variant since it can be mistaken for PTC variants with comparatively poorer prognosis such as tall cell variant, columnar cell variant and poorly differentiated thyroid cancer [7, 11].

In conclusion, this is a rare but an important variant of thyroid carcinoma where both the clinicians and the pathologists should be familiar with. The diagnosis of CMV especially when it is multifocal, warrants ruling out of underlying FAP even in the absence of previously well-established diagnosis of the intestinal polyposis.

## Consent

Written informed consent was obtained from the patient for publication of this case report and any accompanying images.

## Abbreviations

FAP: familial adenomatous polyposis
PTC: papillary thyroid carcinoma
CMV: cribriform morular variant
